# Spatial variation in antler investment of Apennine red deer

**DOI:** 10.1002/ece3.7617

**Published:** 2021-05-03

**Authors:** Stefano Mattioli, Francesco Ferretti, Sandro Nicoloso, Luca Corlatti

**Affiliations:** ^1^ Department of Life Science University of Siena Siena Italy; ^2^ Research, Ecology and Environment Dimensions (D.R.E.Am. Italia) Pistoia Italy; ^3^ Chair of Wildlife Ecology and Management University of Freiburg Freiburg Germany

**Keywords:** allometry, antler investment, deer, life‐history traits, phenotypic quality, spatial patterns, ungulates

## Abstract

Heterogeneity in resource availability and quality can trigger spatial patterns in the expression of sexually selected traits such as body mass and weaponry. While relationships between habitat features and phenotypic quality are well established at broad geographical scales, information is poor on spatial patterns at finer, intrapopulation scales. We analyzed biometric data collected on 1965 red deer *Cervus elaphus* males over 20 years from a nonmigratory population living on two sides of a mountainous ridge, with substantial differences in land cover and habitat quality but similar climate and population density. We investigate spatial patterns in (i) body mass, (ii) antler mass, and (iii) antler investment. We also tested for site‐ and age‐specific patterns in allometric relationship between body mass and antler mass. Statistically significant fine‐scale spatial variations in body mass, antler mass, and, to a lesser extent, antler allocation matched spatial differences in land cover. All three traits were greater in the northern slope, characterized by higher habitat heterogeneity and greater availability of open habitats, than in the southern slope. Moreover, the allometric relationship between body mass and antler mass differed among age‐classes, in a pattern that was consistent between the two mountain slopes. Our results support the occurrence of spatial patterns in the expression of individual attributes also at a fine, intrapopulation scale. Our findings emphasize the role of environmental heterogeneity in shaping spatial variations of key life‐history traits, with potential consequences for reproductive success.

## Graphical abstract

Heterogeneity in resource availability and quality can trigger spatial patterns in the expression of sexually selected traits. In this paper, we investigate spatial patterns in body mass, antler mass, and antler investment in red deer *Cervus elaphus*, one of the iconic game species in Europe. Fine‐scale spatial variations in body mass, antler mass, and, to a lesser extent, antler allocation matched spatial differences in land cover, supporting spatial variation in the expression of sexually selected traits. Photo by Andrea Dal Pian

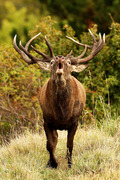

## 1 INTRODUCTION

Spatial variation of environmental factors has a major influence on several ecological processes affecting individuals and populations (e.g., Cromsigt et al., 2009; Karanth et al., 2004; Post et al., 2009). Sexually selected traits, in particular, are sensitive to environmental heterogeneity (Cornwallis & Uller, 2010; Maan & Seehausen, 2011); hence, spatial variation is expected to occur in mating‐related morphological attributes (e.g., insects: Miller & Emlen, 2010; fish: Mollet et al., 2013; amphibians: Lüpold et al., 2017; reptiles: Kwiatkowski & Sullivan, 2002; birds: Møller et al., 2006; mammals: Post et al., 1999). Environmental heterogeneity can occur at multiple geographical scales, resulting in interindividual variation in the expression of morphological traits. In turn, environment‐mediated variation in the expression of sexually selected traits would be expected not only between individuals belonging to different populations (e.g., Kavčić et al., 2020; Lüpold et al., 2017), but also at the intrapopulation scale (e.g., Clutton‐Brock et al., 1982; Miller & Emlen, 2010).

In large herbivores, for example, heterogeneity in key resources can trigger spatial patterns in expression of individual traits at large geographical (e.g., Andersen et al., 1998; Kavčić et al., 2020) as well as at intrapopulation scales (e.g., Clutton‐Brock et al., 1986; Pettorelli et al., 2002). In polygynous ungulates, access to abundant resources is expected to emphasize male investment on sexually selected traits such as body mass and weapon size (e.g., Ashley et al., 1998; Clutton‐Brock et al., 1982; Leblanc et al., 2001; Schmidt et al., 2001). Thus, spatial variation of abundance of key resources would be predicted to elicit spatial patterns of investment on traits such as male horns or antlers.

Given their wide distribution range encompassing a variety of landscapes, and large inter‐ and intraspecific variation in body mass, male cervids are particularly suitable to investigate spatial correlates of individual allocation to sexually selected secondary traits (Geist, 1998). Since their origin in the early Miocene, cervids have been characterized by the presence of deciduous cranial appendages (antlers). Initially, deer lived in tropical and subtropical dense woods, were small‐sized and with relatively small antlers of simple structure, possibly serving as offensive weapons (Geist, 1998). Since the early Pliocene, larger deer species adapted to more open habitats began to appear, with males bearing longer, heavier, and more complex antlers (Croitor, 2018; Geist, 1998; Heckeberg, 2020). Open environments presumably favoured more gregarious behavior and stronger male–male competition for access to mates: Accordingly, size dimorphism and antler size grew in response to more intense sexual selection (Geist & Bayer, 1988; Kitchener, 1991; Pérez‐Barbería et al., 2002). Still today, antlers are relatively larger in cervids forming larger breeding groups and with complex social behavior (Clutton‐Brock et al., 1982; Lincoln, 1992; Plard et al., 2011). Antlers are effective weapons but also honest signals of fighting ability and genetic quality, a conspicuous ornament to threaten other males and attract females (Clutton‐Brock et al., 1980; Geist, 1966; Malo et al., 2005; Morina et al., 2019; Vanpé et al., 2007). With antler investment becoming more demanding and costly, antler development became increasingly dependent on environmental productivity and climate (Brown, 1990; Goss, 1983). At the end of the Early Pleistocene, the first red deer *Cervus elaphus* appeared (Franzen et al., 2000; van der Maden, 1999), characterized by large size and with relatively heavy and well‐branched antlers. Red deer size fluctuated for all Middle and Late Pleistocene and for Holocene in relation to environmental changes (*cf*. Saarinen et al., 2016).

Red deer is among the cervid species with the largest relative antler size (Geist, 1998; Geist & Bayer, 1988). Compared with its more closely related species (sika deer *Cervus nippon* and wapiti *Cervus canadensis*), it has a higher plasticity and can produce a greater relative antler mass under favorable environmental conditions. In low‐productivity habitats, such as Scottish moorlands and Sardinian maquis scrub, red deer are represented by “maintenance phenotypes” (sensu Geist, 1978) with relatively small antlers of simplified structure. Conversely, in rich environments they give rise to “luxury phenotypes,” that is, large‐sized animals with large antlers. For example, Scottish red deer translocated to New Zealand in habitats with superabundant resources have grown heavy and multipointed antlers (Huxley, 1931; Mitchell et al., 1977). Feeding experiments demonstrated that red deer stags weighing 180 kg (prerut live body mass) with 6 kg trophies can produce in three generations 300–350 kg stags with 11–14 kg trophies, if provided with high nutrition planes (Geist, 1986; Vogt, 1947). In central and eastern Europe, some adult red deer stags have attained 320–340 kg of postrut body mass and 17–19 kg of net antler mass (Botev, 1990; Geist, 1986, 1998; Mager, 1941; Szunyoghy, 1959).

Like all highly dimorphic, large, and long‐lived ungulates, the red deer has a prolonged somatic growth, especially in males. Given the high energetic costs to produce skeleton and muscles, males begin to allocate more resources to antlers only when they reach prime age (Gómez et al., 2012). Generally, antlers reach the peak of their development between 8 and 12 years (Drechsler, 1980, 1988; Langvatn, 1986; Mysterud et al., 2005), which approximately coincides with the highest potential reproductive success (Clutton‐Brock et al., 1988; Kruuk et al., 2002; Nussey et al., 2009). To maximize antler mass, adult stags are more efficient than younger stags in mobilizing minerals from the skeleton to support antler growth (Gómez et al., 2012). Antler investment is thus age‐dependent and sensitive to food availability and climate, making antlers reliable indicators of individual quality (Brown, 1990; Peláez et al., 2018).

A strong allometric relationship has been reported between antler mass and eviscerated body mass in adult red deer of different populations (Huxley, 1931). This “positive allometry” is often associated with the growth of conspicuous secondary sexual traits (Kodric‐Brown et al., 2006; O’Brien et al., 2018). Differences in allometric relationship have been observed between subadults (2–4 years old) and adults (aged 5+) (Schröder, 1983). The relationship between antler mass and body mass was also investigated in farmed red deer stags (Ball et al., 1994; Hyvärinen et al., 1977; Moore et al., 1988; Muir & Sykes, 1988) and in other cervid species including white‐tailed deer *Odocoileus virginianus* (McCullough 1982; Jones et al., 2018) and mule deer *O. hemionus* (Anderson & Medin 1965). However, information on how local environmental conditions affect positive allometry is rare for cervids (but see Jones et al., 2018 for white‐tailed deer).

Here, we investigate age‐ and site‐dependent antler investment, body mass, and allometric relationships in a nonmigratory red deer population. We considered two different slopes of an Apennine ridge in Italy with different habitat composition, leading to different productivity. Red deer density and hunting pressure are comparable between the two slopes; there is no supplemental feeding and proximity between sites suggests no major differences in weather, thus allowing to exclude these potentially confounding effects. We hypothesize the local occurrence of a relationship between different morphological features (antler mass, body mass, and antler investment) and age, conditional on sites with different levels of environmental heterogeneity. Namely, we predict that antler mass, body mass, and antler investment will increase up to prime age and then decline in old age (e.g., Drechsler, 1980, 1988), being greater for males in the mountain slope with abundant food‐rich patches (Brown, 1990). Second, we hypothesize age‐specific and spatial heterogeneity in the allometric relationships between body mass and antler mass. Accordingly, we predicted that allometric relationship will vary among age‐classes, possibly increasing with age (Schröder, 1983) and will be weaker in the less productive site (Jones et al., 2018).

## 2 MATERIALS AND METHODS

### 2.1 Study sites

**FIGURE 1:**
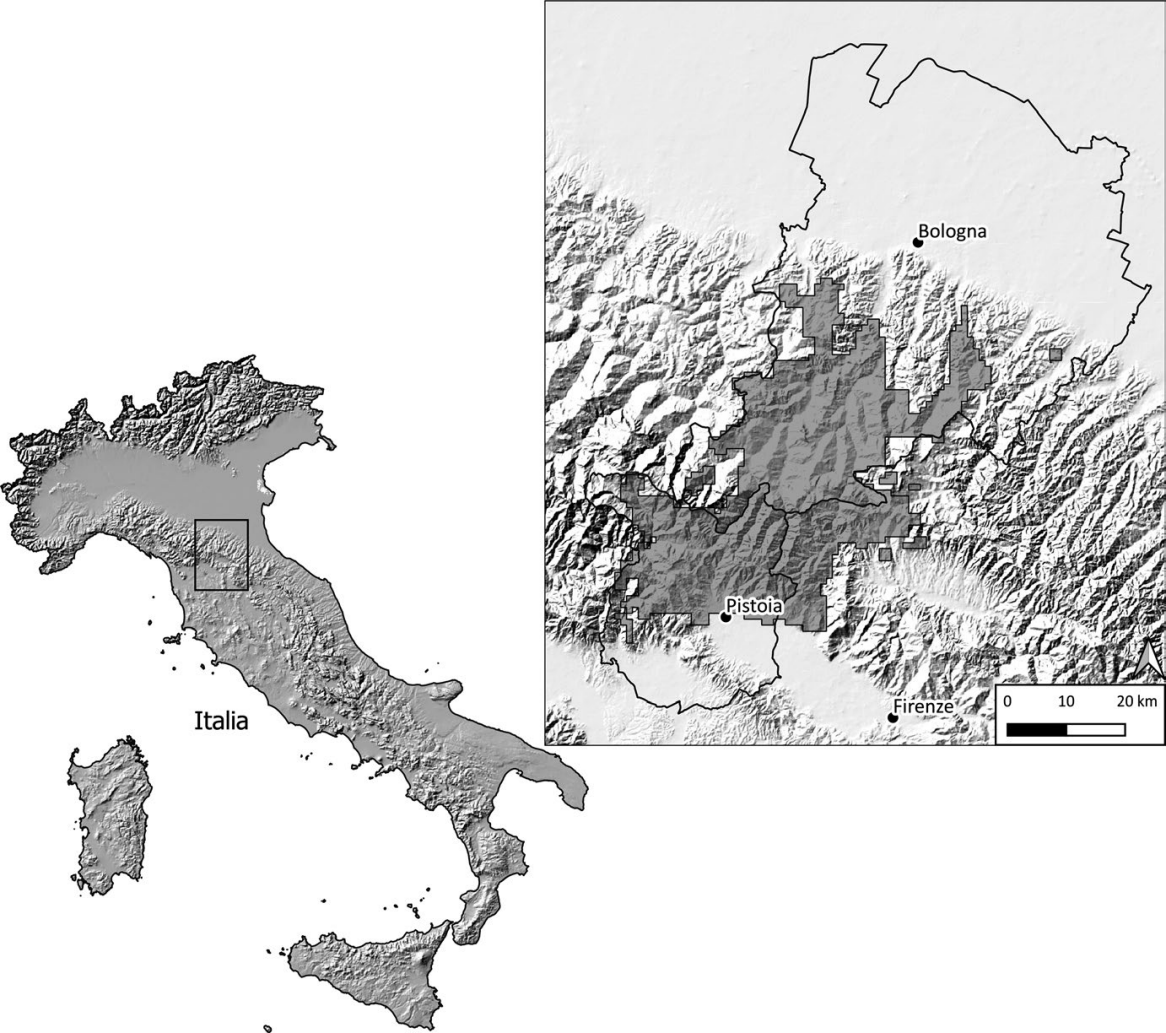
Location of the study area. The panel on the left shows the location of the study area, in the Northern Apennines (Italy). The panel on the right shows the distribution range of the red deer population (gray shaded areas) and the location of the two provinces (Bologna on top and Pistoia on bottom)

The study area (1,400 km^2^) stretches across the two sides of the Apennine chain, at the border between northern and central Italy (Figure 1). The climate is subcontinental cool temperate. Mean annual temperatures range between 9° and 12°C, and mean annual precipitation ranges between 900 and 1,500 mm, mainly owing to altitudinal variations. Winters are relatively mild, with scarce snow fall. The tree vegetation of the hills and low mountains (200–900 m asl) is mainly composed of European hop‐hornbeam *Ostrya carpinifolia*, Turkey oak *Quercus cerris*, pubescent oak *Q. pubescens,* and maples *Acer* spp. and that of the medium‐high mountain (900–1600 m) is primarily composed of beech *Fagus sylvatica*. Plantations of conifers (especially of silver fir *Abies alba* and Douglas fir *Pseudotsuga menziezii*) are uncommon. The landscape and land use of the two slopes are very different (Corine Land Cover 2006: http://www.eea.europa.eu/publications/COR0‐landcover; Table 1). The northern side (province of Bologna) is characterized by higher environmental heterogeneity, with relatively vast forest tracts, small woods, shrubs, meadows, and cultivations: Woods and open habitats cover 52.8% and 39.5% of the red deer distribution, respectively (Table 1). The southern side (province of Pistoia) is mainly made up of large rather compact forests with a few restricted and clumped open habitats (abandoned cultivations, small pastures): woods and open areas cover 80% and 11.5% of the range, respectively (Table 1). Thus, availability of meadows and fields is more than 3 times greater in the northern slope (Bologna) than in the southern one (Pistoia). Moreover, the ratio of the area covered with meadows and fields over the area covered with woodland was 0.65 in Bologna and 0.14 in Pistoia, thereby suggesting higher productivity in the former than in the latter site.

**TABLE 1:** Percentage of major land cover types in the red deer range in the opposing slopes of the Appenine mountains (BO = Bologna and PT = Pistoia)

Land cover types	BO	PT
Cultivated crops and meadows	39.5	11.5
Orchards	–	5.5
Deciduous woods	50.8	73.3
Coniferous woods	2.0	6.4
Shrubs	5.2	0.3
Water (lakes, rivers)	0.3	‐
Urban areas and roads	2.2	3.0

Red deer were reintroduced to the area in 1958–1965 with animals of Alpine stock (Mattioli et al., 2001). Counts were performed since 1994, and the population has been hunted since 2000. Population density in spring is maintained at about 2 ind./km^2^, and no supplemental feeding is provided. The area is also inhabited by wild boar *Sus scrofa*, roe deer *Capreolus capreolus*, fallow deer *Dama dama*, and wolves *Canis lupus*.

### 2.2 Data collection

Antler investment has been largely studied through the ratio of antler size over body size. Geist (1987, 1998) and Geist and Bayer (1988) analyzed the relative antler size (in terms of g of gross antler mass per kg of “metabolic body mass,” that is the postrut live body weight raised to the power of 0.75 and 1.35) of adult red deer and many other deer species to compare the antler investment within the Cervidae. For antler mass data, Geist (1998:183) only used data from exceptionally large‐antlered males (so‐called trophy‐sized males), which he considered more biologically meaningful for taxonomic purposes. Gómez et al. (2012) studied the ratio of antler mass to the skeletal mass in three age‐groups of farmed red deer (yearlings, subadults, and adults 5 years old). Antler mass relative to body mass has been used also in white‐tailed deer (McCullough 1982; Jones et al., 2018), mule deer (Anderson & Medin, 1969), and pampas deer *Ozotoceros bezoarticus* (Ungerfeld et al., 2011). Antler size‐to‐body size ratio has been investigated also using antler length (length of the main antler beam) instead of antler mass (moose *Alces alces*: Stewart et al., 2000; reindeer *Rangifer tarandus*: Melnycky et al., 2013; see Gould, 1973; Clutton‐Brock et al., 1980; Plard et al., 2011; Lemâitre et al., 2014 for reviews on cervid family). Bubenik (1985) proposed as a measure of antler size the total length of the main beam and of all tines. To analyze antler size in their surveys on cervid species, Lemâitre, Vanpé, et al. (2014) and Ceacero (2015) used data on both mass and length.

Here, consistent with most literature on red deer, we adopted the ratio of antler mass over body mass, which quantifies better than other measurements antler investment in terms of efforts to build conspicuous secondary sexual traits. Data on body mass and gross antler mass (mass of upper skull plus antler mass) were collected for 1965 red deer stags legally shot in the study areas (*n* = 1,451 in Bologna; *n* = 514 in Pistoia) between August and March, 2000 to 2019. For each animal, day of harvest and harvest location (hunting district) were recorded. Whole mass and eviscerated body mass of all freshly hunted animals were measured in check stations by technicians and specially trained hunters (Mattioli, 2019), with an electronic scale to the nearest 0.1 kg. Although whole body mass can be affected by rumen content, it was used in this study instead of eviscerated mass because of the difficulty to guarantee uniform dressing of the carcasses and because whole mass is biologically more meaningful than the eviscerated mass to evaluate antler investment (*cf*. Geist, 1998). Whole and eviscerated mass values, however, strongly and positively correlated (Pearson's *r* = 0.98). Gross antler mass (antlers with the whole cleaned upper skull) was weighed to the nearest g after 3 months from culling (dry gross antler mass). The exact age of a subsample of 207 red deer was assessed by counting cementum layers on the inner incisive and, from this sample, a visual guide (De Marinis, 2015) was developed to calibrate estimates from tooth eruption and wear. For the remaining individuals, age estimation was conducted by following carefully the visual guide. Condylo‐basal length of the skull (hereafter “skull length”) was measured with a digital caliper following von den Driesch (1976), to the nearest 0.1 mm. Given the absence of selective criteria in harvest guidelines and the scarce opportunity to encounter and shoot red deer because of the low density, we assumed that hunters did not select animals, and thus, the sample was considered representative of the whole population.

### 2.3 Statistical analysis

To investigate age‐specific variation in antler mass, body mass, and antler investment and its potential difference between study sites, three distinct generalized additive mixed models (GAMMs) were fitted assuming a Tweedie conditional distribution, which generalizes many exponential dispersion models and can handle a wide range of data types, continuous or discrete (Dunn & Smyth, 2018). The response variables “antler mass,” “body mass,” and “antler investment” were thus assumed to be a nonlinear function of age in different study sites (Bologna versus Pistoia). Year of hunting and hunting district were fitted as random intercepts to account for potential differences among hunting seasons and districts. All models were of the general form:

$$E\left({\text{response}}_{\mathrm{ijk}}\right)=\mu_{\mathrm{ijk}}\;\text{and}\;\text{Var}\left({\text{response}}_{\mathrm{ijk}}\right)=\phi \mu_{\mathrm{ijk}}^{p}$$

$$\mu_{\mathrm{ijk}}=f\left({\text{Age}}_{\mathrm{ijk}}\right):{\text{Site}}_{\mathrm{ijk}}+{\text{Site}}_{\mathrm{ijk}}+{\text{Year}}_{j}+{\text{District}}_{k}$$

$${\text{Year}}_{j}∼N\left(0,\sigma_{\text{Year}}^{2}\right)$$

$${\text{District}}_{k}∼N\left(0,\sigma_{\text{District}}^{2}\right)$$

where $\mu_{\mathrm{ijk}}$ was the expected value of the response variable (antler mass/body mass/antler investment) for measure *i* in hunting year *j* and hunting district *k*, *f* the smoothing term for age by site selected via cross‐validation (Wood, 2017), and ϕ the dispersion parameter estimated from the data. In Tweedie models, the conditional distribution is defined by an additional parameter *p* (the Tweedie power parameter): For example, for ϕ = 1, *p* = 0 defines a normal distribution, while *p* = 1 defines a Poisson distribution. The parameter *p* is not constrained to be an integer, and to appropriately model the variance, in this study it was set at 1.5 for the antler mass model and at 1.25 for the body mass and antler investment models, after preliminary inspections of residuals. The random intercepts ${\mathrm{Year}}_{j}$ and ${\mathrm{District}}_{k}$ were assumed to be normally distributed with mean 0 and variance $\sigma_{\mathrm{Year}}^{2}$ and $\sigma_{\mathrm{District}}^{2}$. All models were fitted assuming identity link functions. Therefore, the fitted models essentially reduced to nonlinear mixed models that accommodated the nonconstant variance detected in preliminary analyses and the nonindependence that stemmed from the hierarchical nature of our data.

**FIGURE 2:**
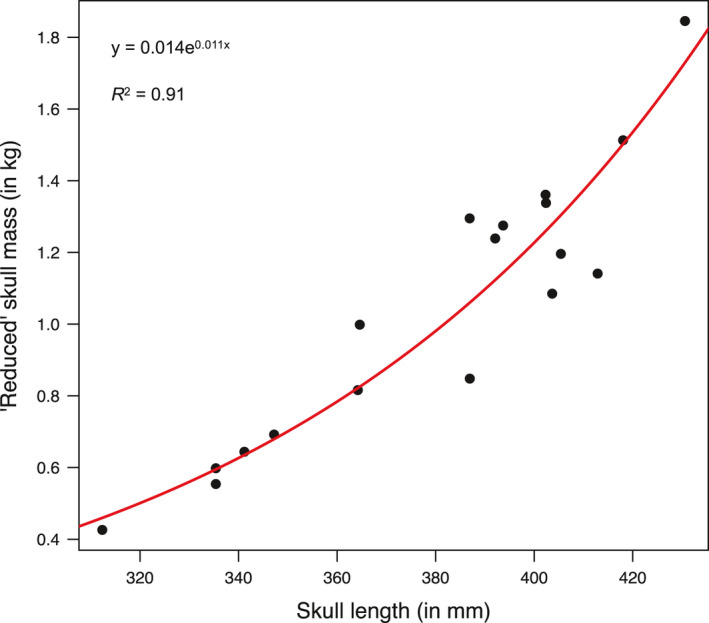
Relationship between skull length and “reduced” skull mass (i.e., antlerless skull mass) estimated from *n* = 18 individuals red deer stags collected in the study sites

The measure of gross antler mass includes skull mass and net mass. With the growing size of antlers, the ratio of the former over the latter tends to decrease. In yearling stags, skull mass may make up more than 70% of gross antler mass; the proportion decreases to *ca*. 40% in 2–4 years old and to *ca*. 25% in individuals aged 5+ years (S. Mattioli and S. Nicoloso, unpublished data). Thus, a proper analysis of age‐dependent antler investment should include net antler mass only (i.e., gross antler mass minus skull mass), to avoid biases related to age‐specific ratio between antler and skull masses. Since net antler mass was not directly available, in the first model an “expected” net antler mass was calculated as the difference between measured gross skull mass (i.e., mass of upper skull plus antlers) and predicted “reduced” skull mass (i.e., mass of upper skull without antlers). For all individuals, the reduced skull mass was predicted from their measured skull length: A small sample (*n* = 18) of antlerless stags independently collected in the same study sites was used to find the function that maximized the *R* ^2^ (0.91) of the relationship between “reduced” skull mass and skull length (Figure 2).

Since animals were hunted between August and March within each hunting season, in the second model whole body mass was adjusted to 20 October (i.e., the end of the rut, which peaks between 20th and 30th September). Mass was adjusted by fitting quadratic linear models between body mass and Julian date from the first day of hunting (10 August) for different age‐classes, because age‐specific trends of mass variation over time are to be expected (*cf*. Radler & Hattemer, 1982; Post et al., 1997). Preliminary quadratic linear models relating whole body mass with Julian date by age‐classes suggested that that age‐classes 1, 2–4, 5–7, and 8+ years (Akaike information criterion [AIC] = −2202) were a better fit than alternate age‐classes 1, 2–7, and 8+ (AIC = −1405) or 1, 2–4, and 5+ (AIC = −2200).

In the third model, antler investment was defined for each individual as the ratio between expected net antler mass and adjusted whole body mass after the rut. For all models, the between‐site differences of smoothed curves were estimated. Notably, we acknowledge that the inspection of tooth eruption and wear might overestimate age in young individuals, and underestimate age in old ones (Gee et al., 2002; Storm et al., 2014). When measurement errors in the explanatory variable are small, compared to the full range of values, this should introduce minor bias in the estimators. However, to investigate more formally the potential consequences of measurement errors, all models were refitted by adding negative random noise (between −2 and 0 years) to the age of young (3‐ to 7‐year‐old) stags and positive random noise (between 0 and +2 years) to the age of old (8+) stags. Model results were consistent between age–data–types; therefore, we decided to keep the original dataset for final inference.

The coefficients of variation of antler investment were calculated for site‐specific age‐classes. Furthermore, we assessed allometric relationships between expected net antler mass and adjusted whole body mass for each age‐class (1, 2–4, 5–7, and 8+ years). Allometric coefficients corresponded to the age‐class‐specific slopes estimated with standardized major axis robust regression models, to account for possible measurement error in both mass metrics, using log‐transformed data (Warton et al., 2006). Allometric relationships were assessed separately for the two populations. Within each population, an age‐class‐specific slope of 1 would suggest isometric relationship, whereas slopes above or below 1 would indicate positive and negative allometry, respectively (Jones et al., 2018).

For all models, goodness of fit was assessed visually through residual diagnostics. All analyses were conducted with R 3.6.1 (R Core Team, 2019) in RStudio 1.2.1335 (RStudio Team, 2019). GAMMs were fitted with the package “mgcv” (Wood, 2017), and their residual diagnostics and marginal effects were investigated with the package “mgcViz” (Fasiolo et al., 2018). For all models, the differences between the values of the site‐specific smoothed curves were investigated with the package “itsadug” (van Rij et al., 2017). The allometric relationships were investigated with the package “smatr” using Huber's M robust estimation (Warton et al., 2012), and their residual diagnostics was investigated with the “stats” package (R Core Team, 2019).

## 3 RESULTS

**FIGURE 3:**
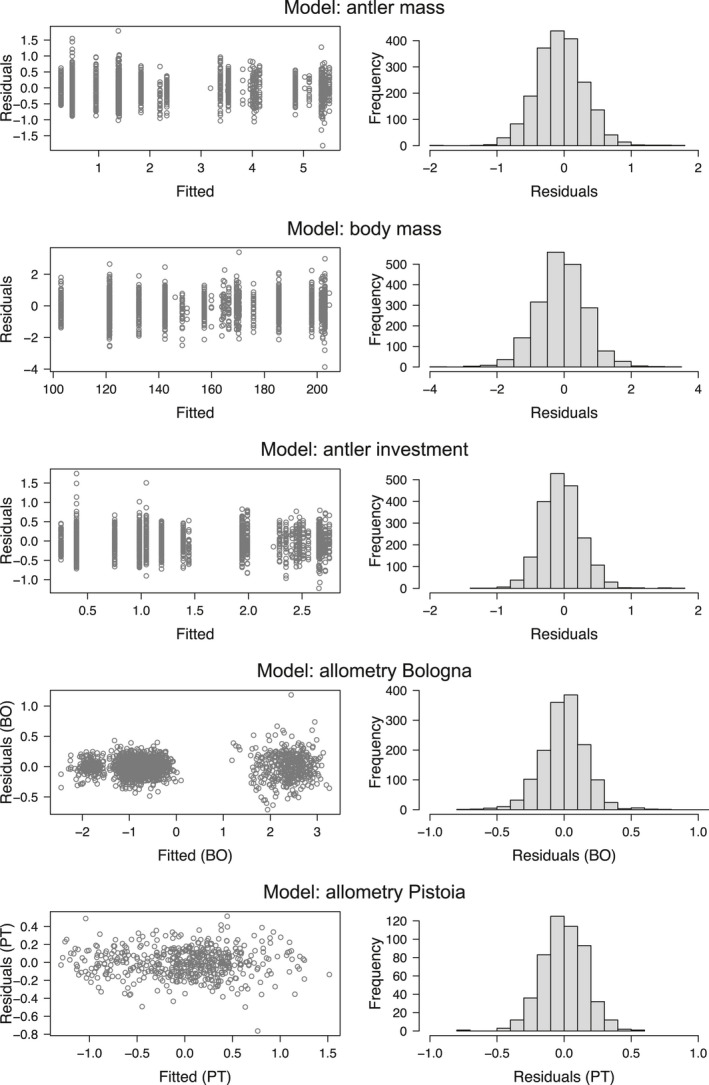
Residual diagnostics (homogeneity of variance on the left and normality on the right) for GAMMs fitted to explain age‐dependent variation in net antler mass, full body mass, and antler investment and for models fitted to explore allometric relationships in red deer in different study sites in the Apennines

**FIGURE 4:**
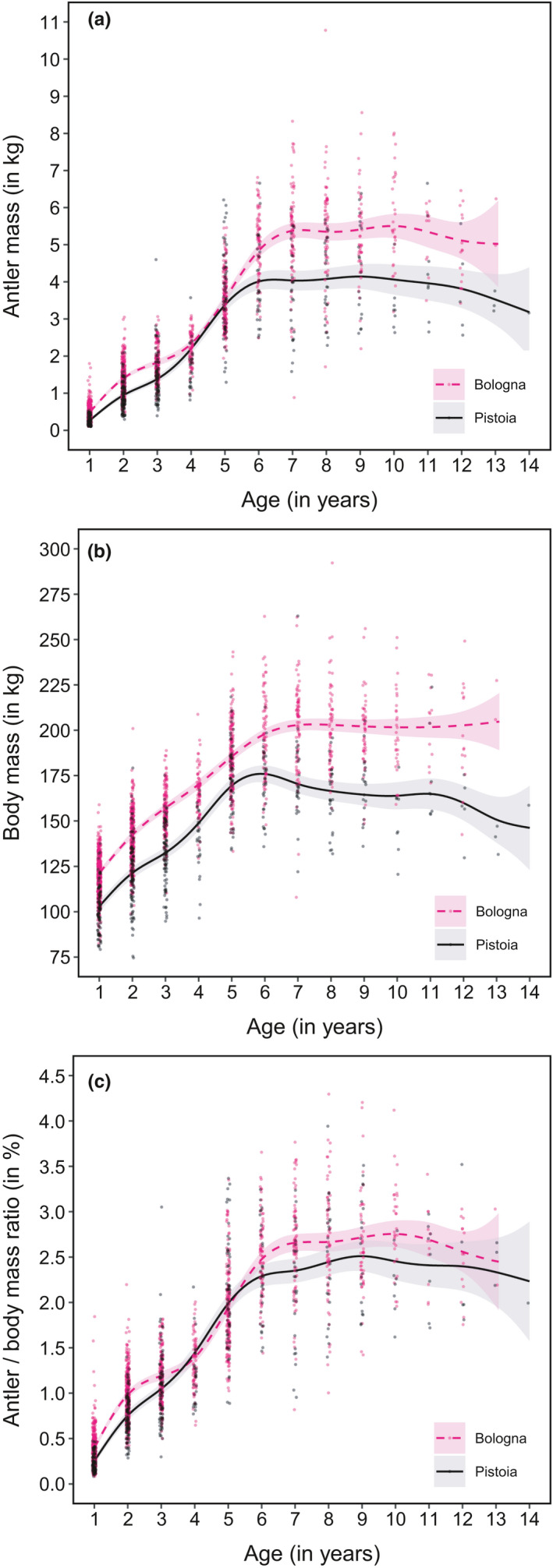
Estimated smoothing curves obtained by GAMMs fitted to investigate the age‐dependent variation in net antler mass (a), full body mass (b), and antler investment (c) in red deer in different study sites in the Apennines. Shaded areas represent 95% confidence interval. Datapoints have been jittered to improve visualization

Residual diagnostics indicated no major violation of assumptions for all models (Figure 3). Mild residual heterogeneity occurred in the estimator for allometry in Bologna (Figure 3), but this should be inconsequential, as the Huber's method used to fit the model offers robustness in inference (Taskinen & Warton, 2011). The GAMMs for antler mass, body mass, and antler investment explained, respectively, about 86%, 77%, and 83% of the variance. The smoothers for different study sites were statistically significant (Table 2), revealing a nonlinear relationship of net antler mass, whole body mass, and antler investment with age. Since GAMM estimates are difficult to interpret, smoothing curves are shown in Figure 4 for all response variables. Notably, preliminary analyses showed that very similar results can be obtained by fitting quasilikelihood GAMMs assuming equality between mean and variance. This supports the goodness of Tweedie models, which were preferred over quasimodels because of lower values of AIC (for quasimodels, the values of AIC were obtained using a wrapper function available in the package “MuMIn” [Bartoń, 2020]).

**TABLE 2:** Estimates of the GAMMs fitted to investigate the age‐dependent variation in antler mass, body mass, and antler investment in red deer in different study sites in the Apennines. The table reports estimates of parametric coefficients (intercept and study site) and estimates of age‐smoothed terms (edf = estimated degrees of freedom)

Parametric coefficients	Estimate	SE	*t*‐value	*p*‐value
Smoothing terms	edf		*F*‐value	
Antler mass				
Intercept	2.430	0.026	93.4	<.001
Site (Pistoia versus Bologna)	−0.517	0.040	−13.0	<.001
s(age) : Bologna	7.036		1,195.5	<.001
s(age) : Pistoia	6.757		486.6	<.001
Body mass				
Intercept	159.368	0.665	239.8	<.001
Site (Pistoia versus Bologna)	−22.992	1.344	−17.1	<.001
s(age) : Bologna	5.943		810.6	<.001
s(age) : Pistoia	6.964		207.4	<.001
Antler investment				
Intercept	1.383	0.017	82.5	<.001
Site (Pistoia versus Bologna)	−0.150	0.022	−6.9	<.001
s(age) : Bologna	7.164		1,091.0	<.001
s(age) : Pistoia	6.507		513.0	<.001

**FIGURE 5:**
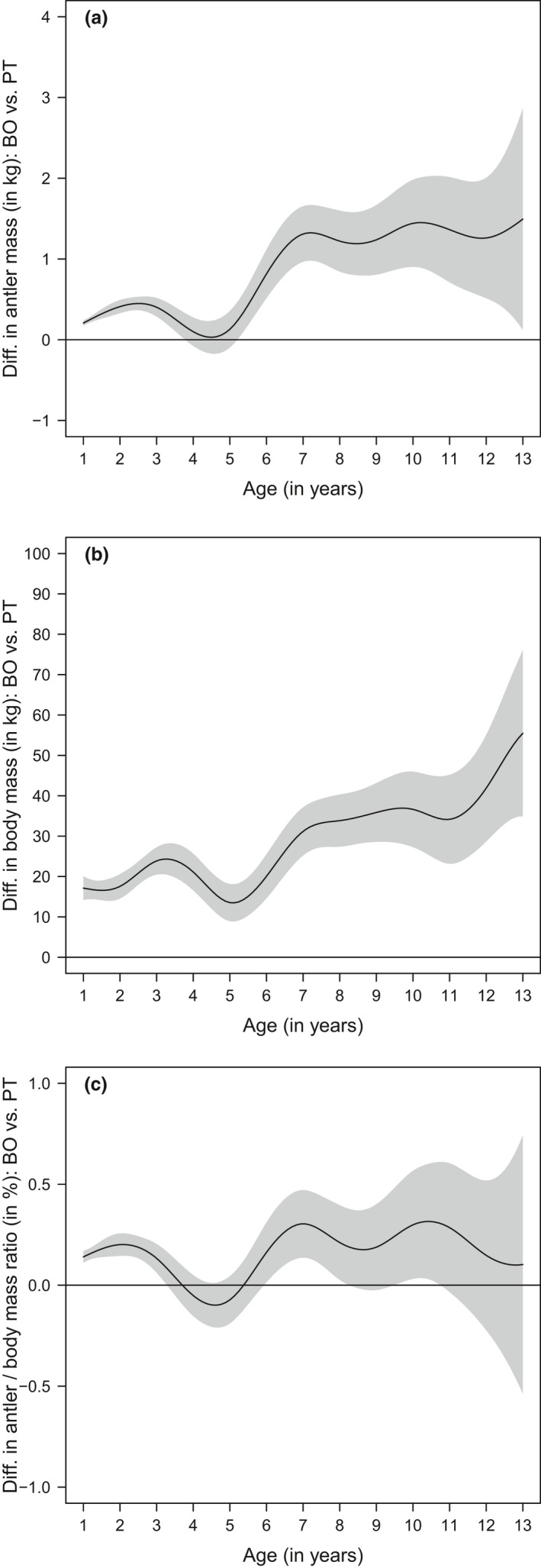
Estimated difference between the values of the smoothed curves for the two study sites (Bologna—BO versus. Pistoia—PT) obtained by GAMMs fitted to investigate the age‐dependent variation in antler mass (a), body mass (b), and antler investment (c) in red deer in the Apennine. The shaded area corresponds to the 95% confidence interval of the difference between smoothed values. When the area does not overlap zero, the values of the fitted curves for the two populations are considered significantly different from a statistical standpoint

Net antler mass increased up to 6 years of age in Pistoia and up to 7 years in Bologna, remained stable until 10–11 years of age, and then appeared to decline (Figure 4a). The greater antler mass observed in Bologna than in Pistoia in the first 3 years of life, and after 5 years of age, was statistically significant (Figure 5a). The mean figure for adult stags (5+ years old) from Bologna was 20.6% higher than from Pistoia (4.65 kg versus 3.83 kg for Pistoia; Table 3). The heaviest recorded antler masses were 10.78 kg for Bologna and 6.66 kg for Pistoia. The CV of antler mass decreased on both side of the Apennine from 48%–51% in yearlings to 29%–31% in adults.

**TABLE 3:** Mean (±*SD*) body mass, antler mass, and antler investment of red deer stags in Bologna (BO) and Pistoia (PT). The table reports body mass adjusted to after the rut (in kg), net antler mass (i.e., whole skull mass ‐ “reduced” skull mass, see text for details), investment (ratio between adjusted body mass and net antler mass), and sample size (*N*)

Age‐class	Postrut body mass (kg)	Net antler mass (kg)	Antler investment (%)	*N*
Yearlings BO	119.4 ± 11.9	0.451 ± 0.228	0.375 ± 0.187	403
Yearlings PT	102.4 ± 12.7	0.245 ± 0.118	0.235 ± 0.099	97
Subadults 2–4 y. BO	148.5 ± 16.7	1.609 ± 0.535	1.074 ± 0.308	541
Subadults 2–4 y. PT	128.6 ± 18.8	1.232 ± 0.607	0.930 ± 0.369	227
Adults 5–7 y. BO	191.8 ± 22.9	4.283 ± 1.242	2.230 ± 0.570	331
Adults 5–7 y. PT	173.4 ± 20.3	3.782 ± 1.136	2.173 ± 0.576	120
Adults 8+ BO	200.1 ± 22.5	5.332 ± 1.248	2.666 ± 0.561	176
Adults 8+ PT	163.3 ± 19.8	4.043 ± 1.131	2.464 ± 0.565	70
Adults 5+ BO	194.7 ± 23.1	4.647 ± 1.339	2.381 ± 0.603	507
Adults 5+ PT	169.7 ± 20.7	3.878 ± 1.138	2.281 ± 0.588	190

Similarly, whole body mass increased up to 6 years of age in Pistoia and up to 7 years in Bologna, but showed a steady decline in the former while remaining stable in the latter (Figure 4b). The heavier body mass observed in Bologna than in Pistoia was statistically significantly for all age‐classes (Figure 5b). Adult stags from Bologna were on average 14.7% heavier than those from Pistoia (194.7 kg versus 169.7 kg; Table 3). The heaviest recorded body masses were 292 kg for Bologna and 263 kg for Pistoia. The coefficient of variation of body mass was approximately constant across age‐classes and in both slopes, at about 10%–15%.

**FIGURE 6:**
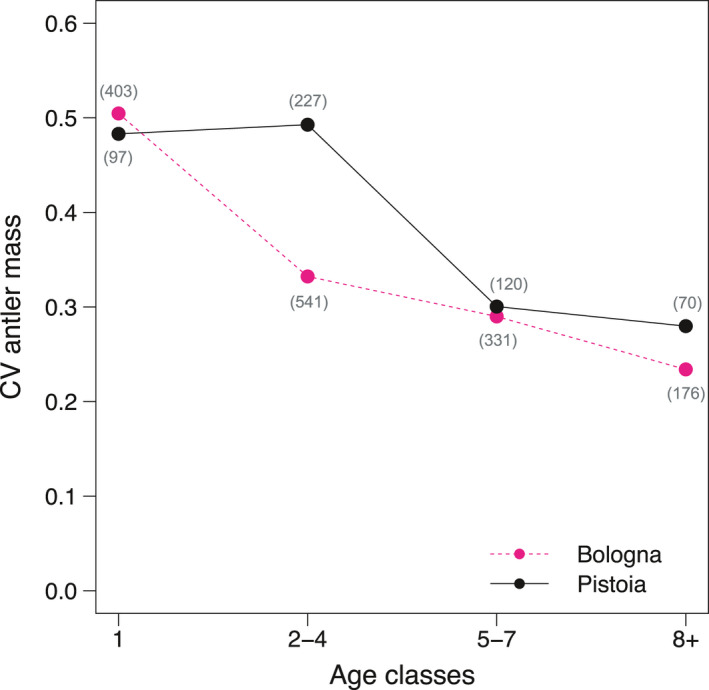
Coefficient of variation in red deer antler investment for different age‐classes in the two study sites. Sample sizes in parentheses

Antler investment increased up to 6 years of age in Pistoia and up to 7 years in Bologna, was stable until 10–11 years of age, and then appeared to decline (Figure 4c). The greater antler investment observed in Bologna than in Pistoia in the first 3 years of life, and between about 6 and 10 years, was statistically significant (Figure 5c). The coefficient of variation decreased with increasing age‐classes in both populations, but the decrease after the first year of age was much sharper in Bologna than in Pistoia (Figure 6). In terms of mean values, antler investment was 0.2%–0.4% in yearlings and increased to 2.5%–2.7% in adults 8+ years old. Mean relative antler mass of prime‐aged stags was 7.1 times greater than that of yearlings in Bologna and 10.5 times in Pistoia. The highest recorded antler investments in adults were 4.3% in Bologna and 3.9% in Pistoia. Yearlings from Bologna had a much higher mean antler investment than those from Pistoia (+59.6%). The CV of antler investment decreased with increasing age‐classes in both populations, from around 40%–50% in yearlings to 25% in adults.

**FIGURE 7:**
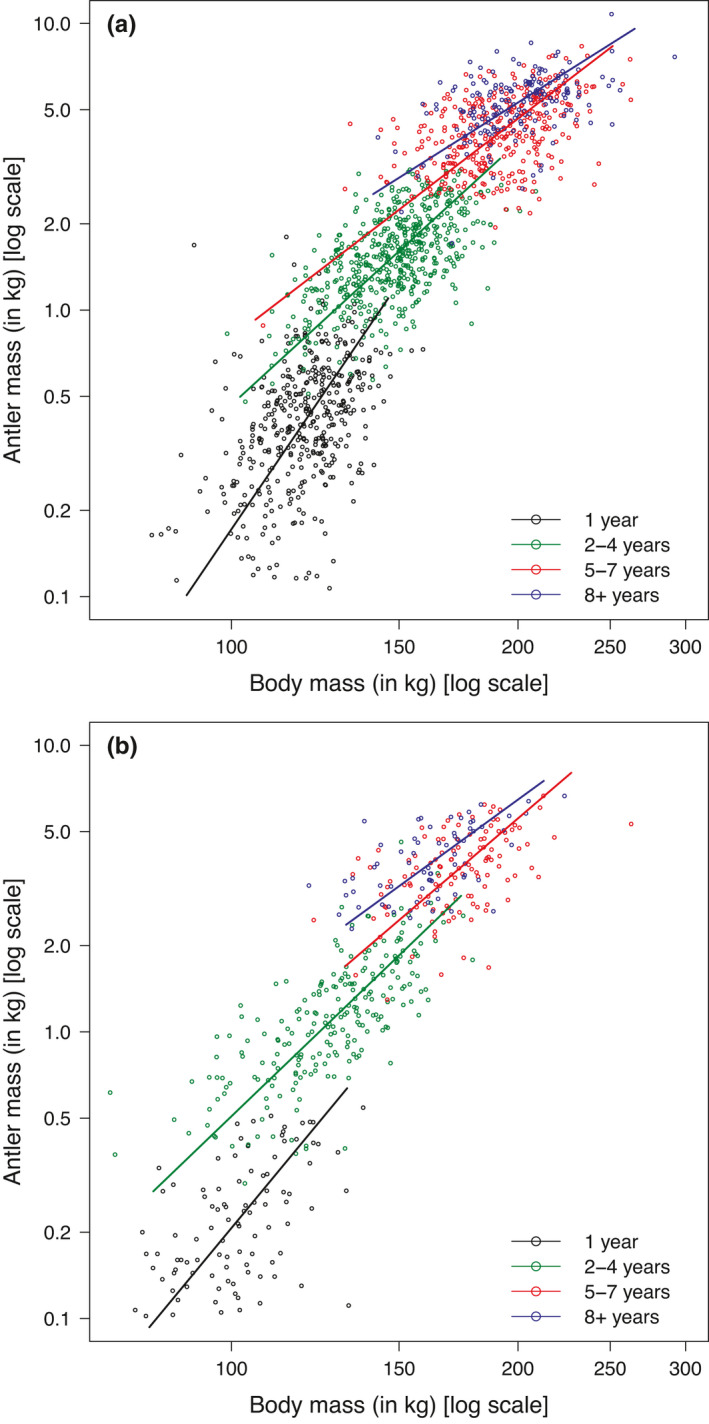
Allometric relationships between net antler mass and adjusted body mass after the rut for different age‐classes (1, 2–4, 5–7, and 8+ years) in Bologna (a) and Pistoia (b)

For both study sites, the allometric relationship between body mass and antler mass was statistically different among age‐classes (Bologna: likelihood‐ratio test [LRT] = 129.3, *df* = 3, *p*‐value <.001; Pistoia: LRT = 17.26, *df* = 3, *p*‐value = .001), although the effect sizes of different age‐class‐specific slopes in Pistoia were broadly more similar than in Bologna (Table 4; Figure 7). All slopes and associated 95% confidence intervals were >1, suggesting positive allometric relationships (Table 4; Figure 7). Generally, allometric relationship was stronger in young stags and weaker in adults over 8 years of age (Table 4; Figure 7). No major differences in age‐class‐specific allometric relationships were observed between sites, with a partial exception for yearlings, which showed a stronger effect size in Bologna than in Pistoia (Table 4; Figure 7).

**TABLE 4:** Allometric relationships between estimated antler mass and adjusted body mass after the rut for different age‐classes (1, 2–4, 5–7, and 8+ years) in Bologna (A) and Pistoia (B). The table reports the site‐ and age‐class‐specific values of sample size (*n*), slope of relationship (slope), lower and upper 95% confidence levels, and *R* ^2^

Study site	Age‐class (in years)	*n*	slope	Lower CL	Upper CL	*R* ^2^
*Bologna*	1	403	4.89	4.45	5.37	0.18
	2–4	541	3.04	2.83	3.27	0.35
	5–7	331	2.54	2.31	2.80	0.21
	8+	176	2.10	1.81	2.44	0.20
*Pistoia*	1	97	4.01	3.40	4.73	0.25
	2–4	227	3.19	2.90	3.51	0.54
	5–7	120	2.84	2.41	3.35	0.22
	8+	70	2.42	2.00	2.92	0.32

## 4 DISCUSSION

We observed remarkable fine‐scale spatial variations in male body mass, antler mass, and antler investment in red deer. These variations matched differences in land cover between two sides of a mountain chain, with heavier and larger‐antlered males on the northern slope characterized by higher habitat heterogeneity and greater availability of open habitats than the southern slope. Accordingly, antler investment of males was also greater in the northern than in the southern side. Moreover, the allometric relationship between body mass and antler mass became weaker in older age‐classes, and it was seemingly stronger in yearling stags from the northern than from the southern side. Our results emphasize the role of environmental heterogeneity in shaping small‐scale variations of key life‐history traits of a highly polygynous species, possibly related to reproductive success (see Pettorelli et al., 2002 for the weakly polygynous roe deer).

As expected for secondary sexual traits of low growth priority, antler investment increased with age and peaked in 8+‐year‐old stags, with a mean production of 26.7 g of antler tissue per kg of body mass in the “rich” side and 24.6 g in the “poor” side. Differences in antler investment between the slopes of the mountain chain were consistently high in yearlings (59.6%), but in prime age they decreased on average to 2.6%–8.2%. It is noteworthy that yearlings from Bologna invested so consistently in their first antler set. They bear mostly simple spike antlers (on average about 40 cm long, but with records of 55–63 cm), but 13% had two or three tines per antler. Yearlings of Pistoia were all spikers, with spikes of approximately the same length but lighter. Possibly, the abundance of food in late spring and early summer was sufficient to support somatic growth and, at the same time, to divert extra‐energies to antler formation. Yearlings are particularly responsive to nutrient availability (Clutton‐Brock & Albon, 1989; Schmidt et al., 2001; Suttie & Kay, 1982), and their body growth and antler size are also influenced by maternal condition and lactation (Gómez et al., 2006). A precocious development of yearlings could exert a strong influence on final adult size, dominance status, and breeding success (Clutton‐Brock et al., 1988). In farmed red deer, body and antler size of yearlings are good predictors of adult size (Moore et al., 1988).

Body mass variability of Apennine red deer stags, as indicated by the coefficient of variation, was consistent across slopes and age‐classes, around 10%–15%, approximately the same as recorded in Mississippi white‐tailed deer (Jones et al., 2018). In contrast, antler mass variability decreased with age, suggesting that as deer approach prime age, they become less sensitive to environmental variation and interindividual differences in antler investment tend to decline. As observed in a study on antler asymmetries (Mateos et al., 2008), red deer stags in prime age appear to converge toward a basic common configuration of their weaponry to enhance fighting ability, thus possibly restraining antler mass variability. While in Mississippi white‐tailed deer average variation in antler mass was lower in the areas with higher nutritional conditions (Jones et al., 2018), in red deer from Apennine differences in food quantity and quality did not appear to play a major role. If adult stags gradually weaken their susceptibility to environmental stressors and interindividual differences in antler mass tend to decrease, this could be connected to the increasing role of skeletal minerals mobilized for antler formation (Gómez et al., 2012; Muir et al., 1987a). When most of the final body size is attained, it is essential for a stag to allocate adequate resources in building strong, symmetrical, and well‐branched antlers (Bartoš & Bahbouh, 2006; Mateos et al., 2008) to successfully compete for access to mating. Antler development becomes a trade‐off between fully expressing their potential, optimizing their functionality, and obtaining minerals from diet and body stores. From a strictly energetic perspective, antler growth of adult stags seems to require relatively modest expenditures, accounting for only 1% of the yearly budget (Bobek et al., 1990), but actually it is a markedly costly activity (Bubenik, 1982, 1985; Dryden, 2016; Moen & Pastor, 1998a,1998b). In a restricted time window, during the 140–165 days of antler formation, and especially between 90 and 110 days from the beginning, adult stags must deposit large amounts of calcium and phosphorus, only partially available from forage (Muir et al., 1987a,1987b); an effort which takes place contemporarily to an increase food intake in preparation for the rut.

With increasing size, antlers face physiological, mechanical, and structural constraints, as suggested by comparative surveys of antler and body size relationships among cervids (Ceacero, 2015; Lemâitre, Vanpé, et al., 2014), but which seems valid also within species (Jones et al., 2018). When approaching the peak of antler development, the largest individuals appear to partly trade antler size for heavy body mass, which can be more determinant in overt contests than longest and heaviest antlers.

We observed a tendency for a decline in antler investment for oldest stags, although our sample included only 26 individuals aged 12–14 years; nevertheless, these results are consistent with those of other studies (Drechsler, 1980, 1988; Langvatn, 1986; Mysterud et al., 2005; but see Nussey et al., 2009 and Lemaître et al., 2014b for the red deer stags of Rum, whose senescence in antler traits was minimal). Tooth wear may impair food assimilation in senescent animals, affecting their body and antler mass.

Our study also shed some light into the complex multiphase positive allometry of body and antler mass, with decreasing exponents from yearlings to older adults. Antler size of Apennine stags continued to increase at a faster rate than body size, but it tended to slow down in prime age, possibly under the influence of physiological and mechanical constraints (Ceacero, 2015; Jones et al., 2018; Lemâitre, Vanpé, et al., 2014). The weaker positive allometry of adult stags at their peak may reflect also the need to adjust the allocation in body mass, which could make the difference in direct fights more than large antlers. Although there are no consistent differences between mountain sides, the degree of overlap of confidence intervals in Table 1 suggests that yearling stags from the most productive site may have a higher allometric exponent than those with a lower nutritional plane. This would indicate a higher antler investment in the former than in the latter, a pattern similar to that observed by Jones et al. (2018) in white‐tailed deer.

**TABLE 5:** Mean antler allocation expressed in g of antler per kg of whole body mass, in prime‐aged males from different red deer populations and other Cervid species

Species/population	Age (years)	g/kg	Source
Red deer, Baranja (H)	8–10	36.5	S. Csányi 2018 pers. com., A. Bokor pers. com. 2020
Red deer, Baranya (HR)	8–10	34.3	Degmečić (2009), modified
Red deer, Apennine (I)	8+	24.6–26.7	This study
Red deer, Carpathians (PL)	9+	22.6	Brewczynski (2002), modified
Red deer, Opole (PL)	7+	19.0	Wajdzik et al. (2018), modified
Red deer, Lower Saxony (D)	8+	17.4	Drechsler (1980), modified
Red deer, Słowinski N. P. (PL)	8+	16.2	Dzięciołowski et al. (1996), modified
Red deer, Mesola Wood (I)	10+	12.2	Mattioli & Ferretti (2014)
Red deer, Rum (UK)	5–10	11.7	Mitchell et al. (1976)
Red deer, Sardinia (I)	5+	11.4	Mattioli & Ferretti (2014), modified
Red deer, Glenfeshie (UK)	5–10	10.2	Mitchell et al. (1986)
Wapiti, Washington (USA)	7–8	34.4	McCorquodale et al. (1989), S. M. McCorquodale pers. com. 1989
Wapiti, New Mexico (USA)	8–10	33.5	Wolfe (1983), L. Bernal pers. com. 2020
Wapiti, Michigan (USA)	9–10	22.1	L. Bender, pers. com. 2020
Common fallow deer, Apennine (I)	5+	28.3	S. Mattioli, unpublished
Common fallow deer (D)	5+	26.0	Siefke & Stubbe (2008), modified
White‐tailed deer, Mississippi (USA)	5–7	11.5	Jones et al. (2018), adapted
European roe deer, Apennine (I)	3+	6.6	S. Mattioli, unpublished
European roe deer (D)	3+	4.4	Stubbe (1990), modified

In a red deer population from Lower Saxony, a two‐phase relationship between subadults and adults was observed (Schröder, 1983), but with a higher scaling exponent for adults. Strict selective criteria applied to the young harvested stags (with higher pressure on low performance individuals) could have influenced the results. In white‐tailed deer, allometric exponents decreased with increasing age‐class until 4 years (Jones et al., 2018); regions with higher environmental productivity were associated with smaller exponents in adult bucks.

For a species typical of open woodland and the interface between forest and meadows (Geist, 1998; Mitchell et al., 1977), the rural landscape of the northern side of the Apennine is relatively more suitable than the southern one. Also in SW Poland, forest districts with a lower wood cover have relatively larger stags with slightly heavier antlers (Wajdzik et al., 2018). In Norway, the proportion of meadows within each municipality had a positive effect on red deer body mass (Mysterud et al., 2002). The gradual closure of the wood after the abandonment of mountain rural economy has negatively affected the productivity of the southern side. Conversely, the greater habitat heterogeneity of the northern side, with woods, shrubs and open areas evenly distributed, provides a higher availability of various food resources. Nevertheless, on a continental scale, body and antler size of red deer living on either side of the Apennine appear relatively high, suggesting locally favorable environmental conditions (especially mild winters) and suitable nutritional conditions, emphasized by the low population densities . During the 1980s, this red deer population had an overall density <1 individual/km^2^; moreover, open grasslands and fields were relatively more abundant. In turn, stags of this population were known for their extremely branched antlers (up to 26–32 tines per pair) and for the high incidence of palmation (20%) (Mattioli, 2003). Overall, this finding confirms the high plasticity in antler growth of this species, whose mean net antler tissue production can range from around 10 g/kg BM in low productive habitats to 40 g/kg BM and more in the most productive ones (cf. Table 5). The highest mean figures are attained in the Pannonian fertile plains of Hungary and in the open woodlands of Carpathian and Balkan Mountains of Romania and Bulgaria (Botev, 1990; Geist, 1998; Szunyoghy, 1959). The highest values recorded in Apennine (39 g/kg BM for Pistoia and 43 g/kg BM for Bologna) are close to the mean values for eastern European countries. The highest values documented for the species in wild conditions are around 50–55 g/kg BM (17–19 kg of net antler mass for a postrut maximum body mass of 340 kg) (*cf*. Geist, 1998).

Our results emphasize the importance of environmental heterogeneity in promoting interindividual variability in the investment in sexually selected traits (e.g., Cornwallis & Uller, 2010; Mann & Seehausen, 2011). While we focused on age‐specific and spatial components, further work would be required to explore temporal heterogeneity (Cornwallis & Uller, 2010), also in relation to changes in climatic and landscape features. Moreover, our results may provide insights into the relationships between investment in sexually selected traits, mating system and sexual size dimorphism. In fact, a comparative evaluation of antler investment in the Cervidae family would help evaluating the role of sexual size dimorphism, mating tactic, and sexual selection in shaping antler investment, which indicates an increasing allocation with growing sexual size dimorphism (Geist & Bayer, 1988; Plard et al., 2011). For example, the roe deer, a weakly dimorphic, territorial species, has the lowest value of antler tissue production (ca 4–7 g/kg BM). White‐tailed deer have a relatively modest sexual size dimorphism, a tending mating tactic (Airst & Lingle, 2019; Hirth, 1977), and show a greater value of antler investment than roe deer. Red deer, wapiti, and fallow deer are highly dimorphic ungulates showing harem defense (or equivalent mating tactics) and have among the highest values of antler tissue production. In conclusion, for polygynous ungulates antler investment, that is, the net production of antler tissue relative to postrut whole weight, can be used as a measure of physical performance of prime‐aged males, with the potential for assessing ecological correlates of a key life‐history trait related to individual reproductive success.

## CONFLICT OF INTEREST

We have no competing interests.

## AUTHOR CONTRIBUTION

**Stefano Mattioli:** Conceptualization (equal); Data curation (equal); Investigation (lead); Methodology (equal); Resources (lead); Supervision (lead); Writing‐original draft (lead); Writing‐review & editing (equal). **Francesco Ferretti:** Conceptualization (equal); Investigation (equal); Methodology (equal); Supervision (equal); Writing‐original draft (equal); Writing‐review & editing (equal). **Sandro Nicoloso:** Conceptualization (equal); Data curation (equal); Investigation (equal); Methodology (equal); Resources (equal); Supervision (equal); Writing‐original draft (supporting); Writing‐review & editing (supporting). **Luca Corlatti:** Conceptualization (equal); Data curation (equal); Formal analysis (lead); Investigation (equal); Methodology (equal); Writing‐original draft (equal); Writing‐review & editing (equal).

## Data Availability

Data used in this analysis are available at Dryad Digital Repository: https://doi.org/10.5061/dryad.37pvmcvk7
